# Rank and Order: Evaluating the Performance of SNPs for Individual Assignment in a Non-Model Organism

**DOI:** 10.1371/journal.pone.0049018

**Published:** 2012-11-20

**Authors:** Caroline G. Storer, Carita E. Pascal, Steven B. Roberts, William D. Templin, Lisa W. Seeb, James E. Seeb

**Affiliations:** 1 School of Aquatic and Fishery Sciences, University of Washington, Seattle, Washington, United States of America; 2 Gene Conservation Laboratory, Alaska Department of Fish and Game, Anchorage, Alaska, United States of America; University of Jaén, Spain

## Abstract

Single nucleotide polymorphisms (SNPs) are valuable tools for ecological and evolutionary studies. In non-model species, the use of SNPs has been limited by the number of markers available. However, new technologies and decreasing technology costs have facilitated the discovery of a constantly increasing number of SNPs. With hundreds or thousands of SNPs potentially available, there is interest in comparing and developing methods for evaluating SNPs to create panels of high-throughput assays that are customized for performance, research questions, and resources. Here we use five different methods to rank 43 new SNPs and 71 previously published SNPs for sockeye salmon: F_ST_, informativeness (I_n_), average contribution to principal components (LC), and the locus-ranking programs BELS and WHICHLOCI. We then tested the performance of these different ranking methods by creating 48- and 96-SNP panels of the top-ranked loci for each method and used empirical and simulated data to obtain the probability of assigning individuals to the correct population using each panel. All 96-SNP panels performed similarly and better than the 48-SNP panels except for the 96-SNP BELS panel. Among the 48-SNP panels, panels created from F_ST_, I_n_, and LC ranks performed better than panels formed using the top-ranked loci from the programs BELS and WHICHLOCI. The application of ranking methods to optimize panel performance will become more important as more high-throughput assays become available.

## Introduction

Molecular markers are widely used in the fields of ecology, evolution, and resource management [1], [2]. Among the many types of markers, single nucleotide polymorphisms (SNPs) have received increased attention due to their potential value for the study of non-model organisms [3], [4]. Their use in ecology and conservation has been demonstrated for several species including mammals, birds, fish, and insects (for example [5], [6], [7]). Additionally, SNPs are abundant throughout the genome; some SNP technologies are robust and automated, enabling accurate and high-throughput genotyping of thousands of individuals [4], [8].

The use of high-throughput SNP panels for the study of non-model organisms has primarily been limited by the cost and difficulties of discovering new SNPs, and consequently, the number of available assays has been low or nonexistent for many species. However, technological advances and innovative methodologies are enabling rapid SNP discovery [9], [10]. With decreasing technology costs [11], SNP discovery projects are becoming more common, and the number of novel SNPs potentially available for conversion to high-throughput assays is rapidly growing (for example [10], [12] and many others).

Population studies in non-model organisms that used high-throughput assays for SNPs typically went through an initial discovery phase where every new assay was precious and every available marker was used (e.g., [13], [14]). Increasingly, many researchers are interested in developing SNP panels of 48 or more that are tailored to their specific research question [15] and study system [13]. Panels of SNPs can be developed and optimized for laboratory performance (i.e. genotypes are easily distinguishable and reproducible), for genotyping platform, and for power to resolve population structure [16], [17]. One approach for identifying loci with high information content for a panel has been to evaluate their ability to elucidate population structure [18]. Additionally, locus selection programs such as WHICHLOCI [19] and BELS [20] are used to rank and evaluate loci based on their performance for individual assignment and in some cases mixed stock analysis (e.g. BELS) [21]. However, there is some concern that upward bias in a SNP's rank can be introduced when using these programs with high-resolution loci [22]. Upward bias is potentially introduced because high-resolution loci are often both discovered and evaluated using the same data set. Although there is currently no consensus on how to rank molecular markers, especially SNPs, ranking and evaluating a SNP's value for a panel will be of increasing importance as the number of high-throughput assays continues to grow.

In sockeye salmon (*Oncorhynchus nerka*) this is already the case. At present, a limited set of 45 SNPs provides insight into life history, migration, and harvest [23], [24], [25]. However, the cultural and economic importance of this species across the Pacific Rim has increased demand for resolving power and created a need for more SNPs and higher resolution SNPs to increase population resolution. In the Pacific Northwest of the U.S., where some stocks are currently listed for protection under the Endangered Species Act, more SNPs are needed to improve resolution of stock structure and provide new options for conservation and management [14]. In Bristol Bay, Alaska, the location of the world's largest fisheries for sockeye salmon, stakeholders seek improved SNP panels to better differentiate among stocks (c.f. [25], [26], [27]). SNPs are also increasingly used for unraveling the complexity of distribution and migration patterns on the high seas [28], [29]. New SNPs and ranking methods will be important for answering these questions and for the management of this valuable resource.

Our objective was to develop SNP panels that could provide improved resolution of sockeye salmon populations inhabiting Bristol Bay as well as provide additional information for studies of migration of mixed populations [29]. Here we both develop new high-throughput SNP assays for sockeye salmon and explore different ranking methods for these and all other SNP assays commonly in use. We successfully developed 5′-nuclease assays for 43 new SNP loci using next generation sequence (NGS) data and high resolution melt analysis (HRMA [30], [31]). These new assays increase the number of published markers for sockeye salmon to well over 100. Additionally, we explore five different ranking methods to evaluate all of these loci: locus-specific values of F_ST_ [32], informativeness (I_n_ [33]), average contribution of a locus to principal components (LC), and locus-ranks from the programs BELS [20] and WHICHLOCI [19]. The ranks from each method were used to create 48- and 96-SNP panels to take advantage of base 48 array platforms commonly in use (e.g. [34], [35]). Panels were then tested for performance using empirical and simulated datasets [36]. All 96-SNP panels, except for the BELS panel, performed similar to one another and better than the 48-SNP panels. Among the 48-SNP panels, panels created from F_ST_, I_n_, and LC ranks performed better than panels using the top-ranked loci from the programs BELS and WHICHLOCI. As more SNPs become available, the differences between methods may have a greater impact on panel performance, warranting careful exploration of locus ranking and evaluation.

## Materials and Methods

### SNP discovery

**Figure 1:**
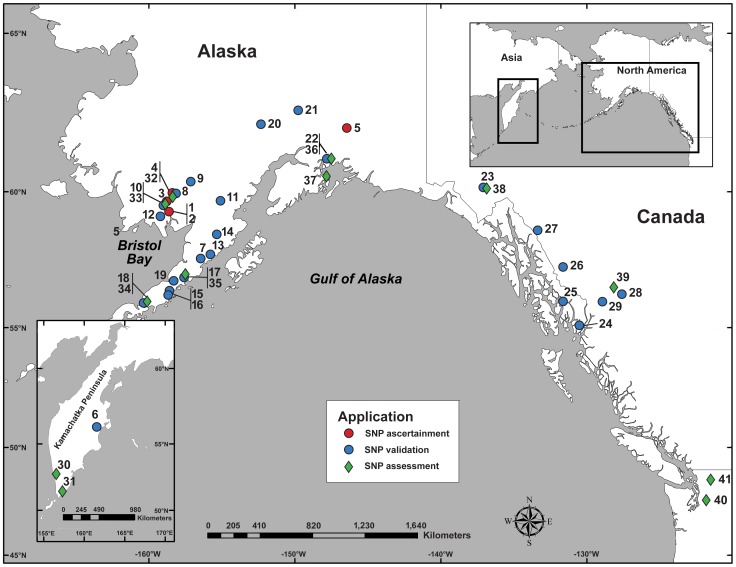
Locations of samples collected for SNP discovery and panel assessment. See Table 1 for location names corresponding to numbers. Sockeye salmon collected for SOLiD sequencing and initial SNP ascertainment [38] are marked with red circles. Samples collected for SNP validation are marked with blue circles. Collections used for SNP assessment and ranking at all 114 SNP loci are marked with green diamonds.

Discovery methods were iterative and adapted for different transcriptome datasets as they emerged from our laboratory. First, primers were selected directly from chum salmon (*O. keta*) 454 assemblies [37]. Additional SNP primers were selected from SOLiD sequence assemblies from sockeye salmon [38]. These latter sequences originated from 10 fish from five locations (Figure 1 red circles; Table 1).

**Table 1:** All collection location and sample sizes sorted by application.

Application	Region	Map #	Location	n
SNP discovery	Bristol Bay, Alaska	1	Yako Creek	2
ascertainment		2	Yako Beach	2
		3	Silverhorn Bay Beach	2
		4	Lake Kulik	2
	Southcentral Alaska	5	Mendeltna Creek	2
SNP discovery	Kamchatka Peninsula	6	Hapiza River	8
validation	Bristol Bay, Alaska	7	Deer Creek	8
		8	Tikchik River	8
		9	Upper Nushagak-Klutapuk Creek	8
		10	Pick Creek	8
		11	Upper Talarik Creek	8
		12	Ualik Lake tributary	8
		13	Becharof Creek	8
		14	Margot Creek	8
	Alaska Peninsula	15	Hatchery Beach, Chignik	8
		16	Broad Creek	8
		17	Cinder River	8
		18	Bear Lake	8
		19	Meshik River	8
	Southcentral Alaska	20	Yentna River slough	8
		21	Susitna River slough	8
		22	Coghill Lake	8
	Southeast Alaska	23	Klukshu River, Alsek	8
		24	Hugh Smith Lake	8
		25	McDonald Lake	8
	British Columbia, Canada	26	Scud River	8
		27	Taku River mainstem	8
		28	Slamgeesh Lake	8
		29	Meziadin Lake Beach	8
SNP assessment	Kamchatka Peninsula	30	Bolshaya River	90
		31	Ozernaya River	93
	Bristol Bay, Alaska	32	Lake Kulik	68
		33	Pick Creek	84
	Alaska Peninsula	34	Bear Lake	93
		35	Cinder River	89
	Southcentral Alaska	36	Coghill Lake	89
		37	Main Bay	61
	British Columbia, Canada	38	Upper Tatshenshini River	88
		39	Damdochax Creek	85
	Washington	40	Issaquah Creek	87
		41	Baker Lake	93

Primers were designed and tested for PCR amplification of a single product on a single pooled sample of DNA. Successful primers were then used to screen individuals for SNPs using HRMA as in McGlauflin et al. [31]. HRMA was performed following the manufacturer's instructions on Lightcycler 480 (Roche Diagnostics) platform using eight test fish from each of 24 locations (192 fish total; Figure 1 blue circles; Table 1). These locations were chosen to focus upon Bristol Bay populations and also include a few representatives from the eastern and western Pacific Ocean.

Putative SNPs that were successfully detected using HRMA were selected for Sanger sequencing. Sequences where the identity of the SNP was confirmed by the presence of at least two genotypes were used for designing primers and probes for the 5′-nuclease assays. As a final validation step, each assay was then tested by genotyping the same panel of 192 fish that were used for HRMA. Assays that did not perform well or where the SNP deviated from Hardy-Weinberg expectations (HWE) were discarded (HWE was tested on a subset of populations for which we possessed additional samples of (N = 61–95). The Sanger sequences used for 5′-nuclease assay design were used to annotate validated markers using the NCBI sequence database and Blastx. Only assays where the most similar sequence hit had an e-value <1.0E-10 were annotated.

### SNP assessment

Six pairs of population samples (hereafter referred to as assessment populations) were chosen from throughout the species' range to assess within and among region variability (Figure 1 green diamonds; Table 1). All fish from the 12 assessment populations were genotyped at 114 nuclear loci (Table S1) using 5′-nuclease assays [34]. These SNPs included the 43 new SNPs described in this paper, 68 previously published SNPs for sockeye salmon [14], [29], [39], [40] and three unpublished markers from the Department of Fisheries and Oceans Canada (Molecular Genetics Laboratory, Pacific Biological Station, Department of Fisheries and Oceans Canada).

Tissues (heart, liver, fin, or axillary process) or genomic DNA were obtained from archived samples at the University of Washington (UW), the Alaska Department of Fish and Game (ADF&G), and the Washington Department of Fish and Wildlife (WDFW). Genomic DNA was extracted as necessary using the DNeasy96 Blood and Tissue Kit (QIAGEN, USA).

The markers were first evaluated using standard population genetic indices using the 12 assessment populations (Table 1) as follows. Populations were tested for deviations from HWE at each locus using chi-square tests as implemented in GenAlEx 6.2 [41]. All critical values were corrected for multiple comparisons using a sequential Bonferroni correction [42]. Allelic richness was calculated for each locus in each population using FSTAT v.2.9.3.2 [43] to look for effects of ascertainment bias. Differences in average allelic richness among locations were tested for significance with an ANOVA. Linkage disequilibrium was tested in each collection for each pair of SNPs using Genepop 4 [44]. To check for genotyping error, 8% of each collection was genotyped again.

Population differentiation was measured as F_ST_ [32] at each locus using Genepop 4 and between population pairs across all loci using Arlequin 3.5 [45]. A principal coordinate analysis with six coordinates was performed in GenAlEx to visualize the genetic relationship among populations. Arlequin was also used to detect outlier loci, candidates for directional selection [46], across the entire range using the hierarchical island model with six regions (Table 1), 20,000 simulations, 100 demes, and 50 groups. Detection of candidate loci was based on Beaumont and Nichols original work using heterozygosity and high differentiation to identify outlier loci [47]. The value of these outlier loci to resolve populations was investigated by removing these loci from the data set and then re-measuring genetic differentiation between populations. Significance of differences in genetic differentiation measured with outlier loci and without outlier loci was tested using a Mantel test.

### SNP ranking

Each locus was ranked according to five measures: F_ST_ [32], informativeness as calculated by Rosenberg (I_n_ [33]), average contribution of a locus to principal component analysis (LC), BELS ranking [20], and WHICHLOCI [19]. We additionally considered the ranking approach GAFS of Topchy et al. [48]; GAFS was not implemented because of its similarity to BELS and computational costs [17]. Each method used is summarized in Table 2. F_ST_, LC, and I_n_ are all measures of genetic diversity based on differences in allele frequencies observed at a locus, while BELS and WHICHLOCI are scores based on maximizing the likelihood of assigning a genotype to the correct population. Informativeness (I_n_) has been shown to be correlated with F_ST_ by Rosenberg et al. [33]. Informativness's relationship to LC was determined using a Spearman's rank correlation. The LC was determined using a multivariate locus comparison method developed by Moazami-Goudarzi and Laloë [49] and implemented in S-Plus (MathSoft, Inc, 2000). Here, locus contribution was determined for the first five principal components.

**Table 2:** Descriptions of the different approaches used for ranking SNP loci.

Ranking approach	Description	Reference
F_ST_	Scaled among-population variance in allele frequency	Weir & Cockerham 1984
Locus contribution (LC)	Average contribution of each locus to principal components	Moazami-Goudarzi & Laloë 2002
Informativeness for assignment (I_n_)	Estimates potential for an allele to be assigned to one population in comparison to an average population	Rosenberg et al. 2003
BELS	Ranks a locus' performance for maximizing mixture estimation accuracy during individual assignment	Bromaghin 2008
WHICHLOCI	Determines locus efficiency for correct population assignment and propensity to cause false assignment	Banks et al. 2003

BELS and WHICHLOCI provide each locus a rank based on the accuracy of individual assignment for that locus and the value lost when the locus is removed from the panel in a jackknife fashion. Loci that result in the greatest loss in individual assignment performance when removed receive the highest score. Both of these locus-ranking programs were run with resampling for a simulated population size of 200 individuals and with 250 iterations. No critical population was defined. In WHICHLOCI, minimum correct assignment was set at 95.0%. In BELS, the performance measure was designated to maximize mean individual assignment accuracy for 100% correct assignment. For BELS, the role of locus input order was explored by running the analyses with four different locus orders: alphabetical, reverse alphabetical, and two randomly generated locus orders. Differences in locus ranks for each input order were tested in a pairwise fashion using the Wilcoxon Signed Rank test.

Initially, each locus was ranked using all individuals available (full set) for the twelve SNP assessment populations (Table 1). However, to reduce the potential for upward bias introduced when loci are ranked and assessed using the same individuals, Anderson's Simple Training and Holdout method [22] was implemented. Half of each assessment population was randomly selected for locus ranking (training set). For odd numbered population size the extra individual was assigned to the training set. The remaining individuals (holdout set) were reserved for panel testing. Significance of differences in locus ranks using the full population set and the training population set were tested using the Wilcoxon signed rank test.

### Panel testing

SNP panels were designed to assess the value of increasing the number of markers included in a panel and to evaluate the different measures for ranking SNPs using the 12 assessment populations. Two panel sizes were selected, 48 and 96 SNPs, to test for differences in resolving power when the number of markers was increased. These panel sizes represent the capabilities of high-throughput genotyping platforms commonly in use at that time (e.g. [35]).

We assembled seven pairs of 48-SNP and 96-SNP panels. Using the training set, five pairs were created from the top ranked loci for each locus measure. A sixth pair of panels was constructed from top ranked loci (based on their average rank). Finally, a seventh pair of panels was constructed from randomly selected loci.

Each panel was tested for performance with two different methods. Using the program ONCOR [50], assignment tests were performed assigning holdout set individuals from each assessment population (Table 1) to a baseline of the training set individuals that had been used for SNP ranking. Since the origin of assigned individuals was known, the probability of assignment to the population of origin was reported for assignment accuracy. The second method used to assess panel performance was a simulation of individual assignment described by Rosenberg [36] as implemented by Ackerman et al. [51]. These simulations use the allele frequencies for user-described populations to assign a simulated individual back to the correct population and report the probability that this assignment is correct. Here individuals were simulated using allele frequencies from holdout set individuals for each population. For each panel, individual assignment was simulated 500 times with 1000 individuals in R, and the frequency of correct assignment (*f*_ORCA_ [36]) was reported.

Differences in panel performance for both assessment methods were tested for using an ANOVA and the post hoc Tukey's Honestly Significant Difference test (α = 0.01).

In addition to panel testing, we examined the value of using the full set of loci and the change in assignment accuracy with decreasing panel size after the subsequent removal of loci. Beginning with the full set of 110 polymorphic loci, ONCOR was used to determine probability of correct assignment similarly to 96- and 48- SNP panel assessment. Loci were then excluded five at a time by lowest average rank (Table S1) until only the five top ranked loci remained for individual assignment. Mean values and 1^st^ and 3^rd^ quartiles were calculated from the resulting probabilities in Excel (Microsoft for Macs 2011).

## Results

### SNP Discovery

We developed 5′-nuclease assays for SNP genotyping of sockeye salmon from both ascertainment sources: chum salmon contigs originating from 454 assemblies [37] and sockeye salmon contigs originating from SOLiD assemblies [38]. Over 1800 potential primers were initially tested; of these, only 515 passed the initial PCR test (Table 3). This test ensured that PCR amplification would occur and that only one product would amplify. Unsuccessful amplification could be a result of the primer annealing on or near intron-exon boundaries. Templates that did not contain a putative SNP or that contained multiple polymorphisms were eliminated using HRMA. Multiple polymorphisms in the same template were attributed to paralogous sequence variation, known to be problematic in tetraploid-origin salmonids [37]. Putative SNPs derived from SOLiD sequence had a failure rate three-fold higher than that observed in putative SNPs derived from 454 sequence (Table 3). Many of the remaining 148 putative SNPs were polymorphic in the majority of the test populations, and resequencing confirmed the identity of at least two genotypes in 93 of these. We attempted to design 5′-nuclease assays for all 93; only 43 had differentiable genotypes that met HWE (Table 3). Twelve of these validated SNPs were annotated based upon sequence similarity (Table S2).

**Table 3:** Summary of SNP discovery and validation.

		Validation procedure			
Sequence Source	Primer Pairs	PCR test	HRMA	Sanger sequence	5′-nuclease genotype
Chum 454^1^	308	108	71	47	19
Sockeye SOLiD^2^	1536	407	77	46	24
Total	1844	515	148	93	43

The number of primers that amplified a single product are shown for the first validation procedure, PCR test. The number of primer pairs that had melt curves with putative SNPs are shown for HRMA validation. The Sanger sequencing validation procedure shows the number of sequenced HRMA products that confirmed the SNPs identity for a primer pair. SNPs that were successfully genotyped from these sequences in a 5′-nuclease genotype are shown for the final validation procedure.

1 Seeb et al. 2011 [37].

2 Everett et al. 2011 [38].

### SNP assessment

Each fish from the 12 populations used for SNP assessment was genotyped at 114 nuclear loci (Table S1). These were all of the 5′-nuclease assays for sockeye salmon, with reliable laboratory performance, that were available at the time.

Re-genotyping discrepancies were less than 1% in all populations. Individuals missing genotypes at more than 10% of the loci were excluded from analyses. Sample sizes reported in Table 1 are all individuals included in post-genotyping analyses after removal of individuals with missing information. Poor tissue quality, which hampers genotyping ability due to the degradation of DNA, was the most likely cause for the low genotyping success in some fish. Four loci were monomorphic in all 12 populations and were removed from subsequent analyses (Table S1). All remaining 110 loci were retained in the data set.

In four collections, there were deviations from HWE at a single locus after correction for multiple tests: Ozernaya River at *One_zP3b*, Damdochax Creek at *One*_*U1202-105*, Pick Creek at *One*_*Tf_ex3-182*, and Baker Lake at *One*_ *U1102-220*. In each of these collections there was a rare homozygote genotype at each of these loci and average minor allele frequencies less than 0.03 with one exception. There was an excess of heterozygotes in the Baker Lake collection for *One_U1102-220*. Mean allelic richness varied across locations and ranged from 1.8 in Lake Kulik to 1.95 in Main Bay (F = 2.74, P = 0.002). Significant deviation from linkage equilibrium was observed in over half of the collections for only three pairs of loci: *One_aldB-152* & *One_ALDOB-135*, *One_GPDH-201* & *One_GPDH2-187*, and *One_MHC2_190* & *One_MHC_251*. These loci were treated as independent for the remaining analyses because we wanted ranking and panel testing to include all available loci for the species. Retaining these loci in the data set could lead to upward bias in assignment success due to redundant information. However, there are only three pairs of loci which were not in linkage equilibrium, and these loci were not in disequilibirium in all populations, warranting their retention in downstream analyses.

**Figure 2:**
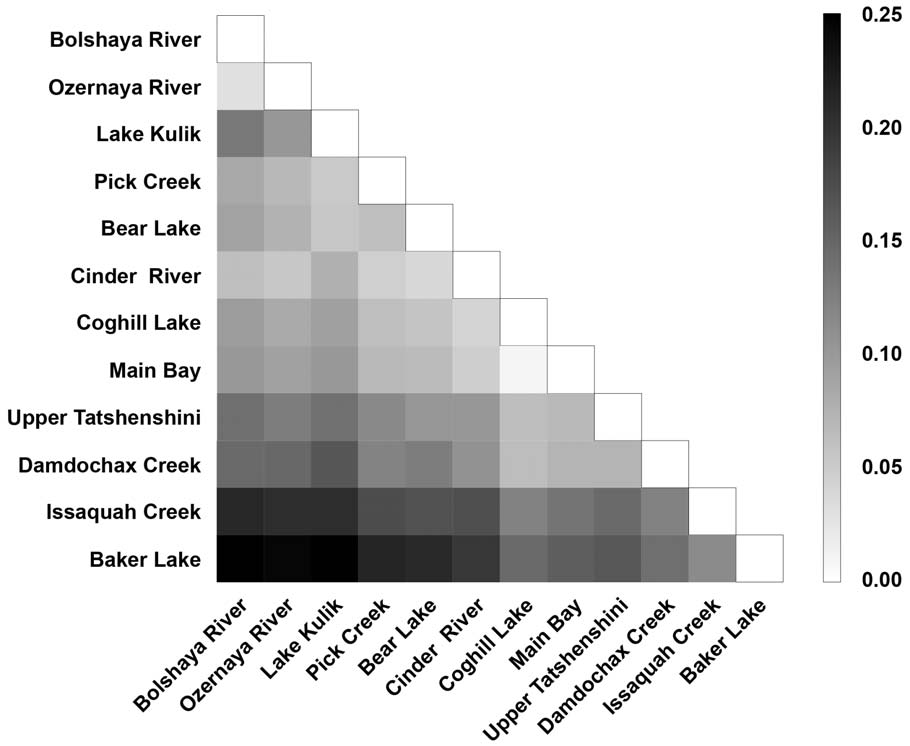
Matrix of pairwise F_ST_ values for all population comparisons. Values calculated for all 110 SNPs. Shading reflects degree of divergence and corresponds to F_ST_ values indicated in legend (right). Populations are in geographic order from Kamchatka to Washington.

**Figure 3:**
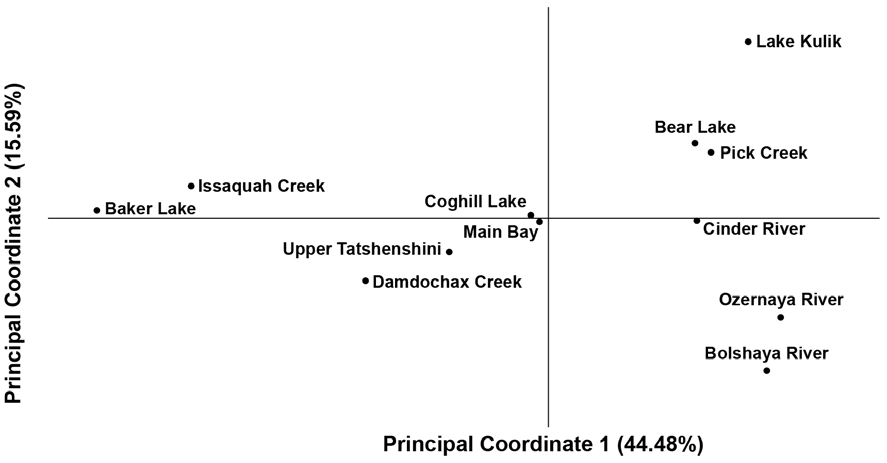
Principal coordinate analysis of SNP assessment populations. The first and second principal coordinates are based on population allele frequencies for 110 SNPs. The percentage of variance accounted for by each coordinate is given in parenthesis.

**Figure 4:**
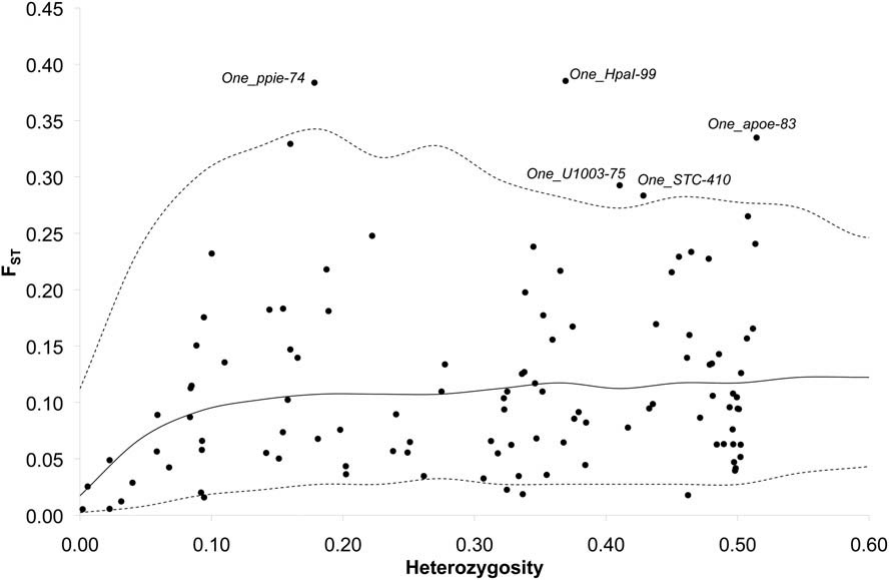
Heterozygosity and F_ST_ for assessment populations. Values were calculated for all 110 SNPs and the upper and lower 99th quantiles are denoted with dashed lines. The 50th quantile is denoted with a solid line. Loci lying outside of the upper 99th are labeled and considered to be candidates for directional selection.

The average F_ST_ was 0.114 for all 110 polymorphic SNPs across all collections (Table S1). There was significant genetic differentiation between all population pairs (P<0.001) except for the pair from Southcentral Alaska (Table 1; populations 36 & 37); however, the level of differentiation between and among regions is variable as indicated by the heat map (Figure 2). The genetic relationships among populations can be seen in the PCA (Figure 3) where the population pairs generally cluster and are separated clinally from east to west on principal coordinate 1 (44.5% of the variation observed among the collections). Population differentiation across the species range may be driven by the five candidate loci (Figure 4). Two of these were new loci described in this paper. When these candidate loci were removed from the data set, the same pattern of genetic differentiation was observed with only the pair of Southcentral populations remaining indistinguishable. However, all F_ST_ values were lower without these outlier loci, and there was a significant difference in genetic differentiation measured when these outlier loci were removed (Z = 0.94, P<0.01).

### SNP ranking

**Figure 5:**
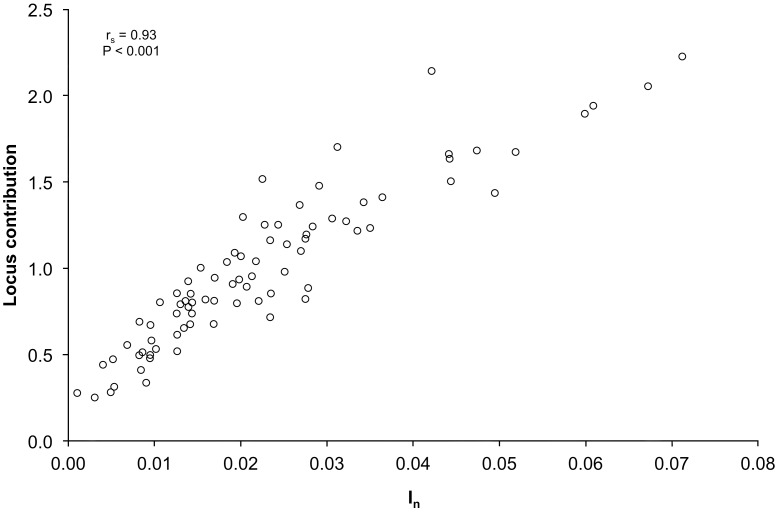
Spearman rank correlation between LC and I_n_. The average contribution of a locus to principal component analysis (LC) and the locus informativeness (I_n_) are calculated for the 12 assessment populations and 110 SNPs.

**Figure 6:**
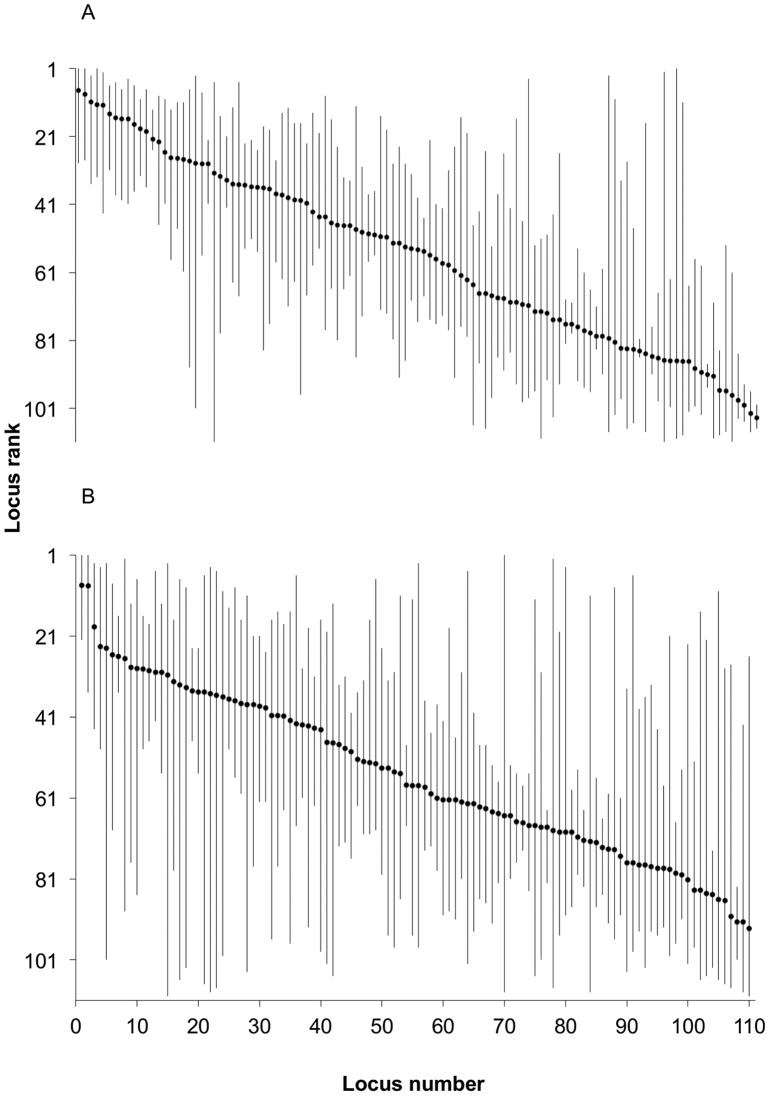
Average rank for all polymorphic loci. Loci ordered from left to right by highest average locus rank (locus number) as in Table S1 for the full 12 assessment populations (A) and for training-set individuals only (B). Average locus rank indicated by closed circles with bars extending from the highest and lowest rank for that locus from the different ranking procedures.

**Figure 7:**
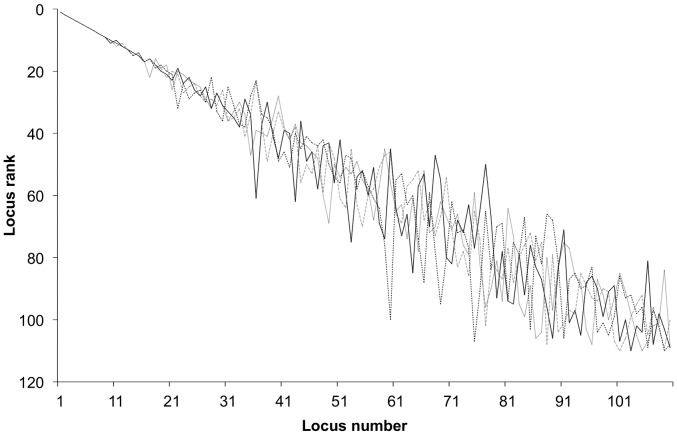
Difference in BELS locus ranks with input order. Input orders: alphabetical (dotted line), reverse alphabetical (solid line), and two randomly generated loci orders (black dashes and grey dashes). Locus number corresponds to average locus rank (Table S1).

Informativeness values (I_n_) were highly correlated with the locus contribution (LC) (r_s_ = 0.93, P<0.001; Figure 5) using a Spearman rank correlation. I_n_ was also highly correlated (r_s_ = 0.99) to F_ST_ as shown by Rosenberg et al. [33]. Most loci were ranked differently using each method for both the full population set and the training set (Figure 6). The greatest differences in rank were observed for loci with small heterozygosities (e.g. *One_gadd45-269* and *One_parp3-170*). Often these loci received a high rank (low number) from BELS and a lower rank (high number) from F_ST_, I_n_, and LC measures (e.g., *One_redd1-414* and *One_serpin-75*). BELS rank did vary with input order (Figure 7), but all of the top-ranked loci remained top-ranked loci and the variation in locus rank was not significantly different between ranks from the different input orders (P = 0.59–0.97).

Although there were differences in locus ranks, the 96-SNP panels contained many of the same loci as there were only 110 loci available. When using the full population set to determine locus rank, only 3–7 loci differed between the five 96-SNP panels created using the different ranking methods (Table 2). Up to 13 loci differed between the five panels when ranks were determined using only the training set, which contained half as many individuals. However, F_ST_ and I_n_ panels shared all but one locus.

There were fewer loci shared between the 48 SNP panels. The F_ST_, I_n_, and LC 48-SNP panels from the full population set had up to 11 different loci, and each of these shared only a little over half of their loci with the BELS and WHICHLOCI panels. The F_ST_, I_n_, and LC panels from the training set were more similar with only 3–7 different loci. Only 16 loci differed between these panels and the WHICHLOCI panel, while the BELS panel had the most unique loci, sharing as few as 12–20 loci with another panel. There was no significant difference in average locus rank (P = 0.96) despite differences in panel composition with two different population sizes (full set vs. training set). Since the purpose of splitting the SNP assessment populations into a training set for SNP ranking and a holdout set for assessing SNP performance was to reduce upward bias only, training set ranks were used for panel testing.

### Panel testing

**Figure 8:**
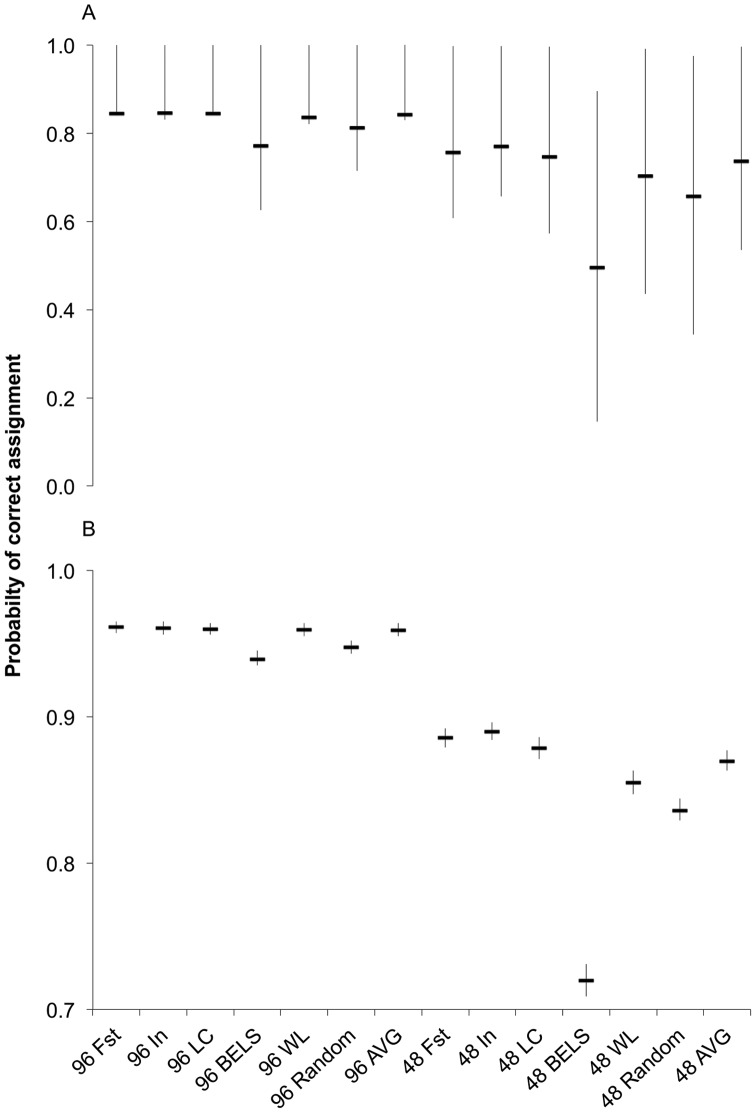
Probability of correct assignment for 48- and 96-SNP panels using empirical data (A) and simulated data (B). Each panel contains the highest ranked loci for each ranking approach: F_ST_, informativeness (I_n_), average contribution of a locus to principal components (LC), the locus-selection program BELS, and the locus-selection program WHICHLOCI (see Table 2). The random panel contains loci chosen at random. Whiskers extend to the 1^st^ and 3^rd^ quartile around the mean.

There was a significant difference in mean assignment scores using empirical (F = 48, P<0.0001) and simulated (F = 27409, P<0.001) data (Figure 8). In the empirical data there was greater variation in probability of correct assignment and fewer significant differences between panel performances (Tables 4, 5). The average probability of correct assignment for empirical data was 0.83 for the 96-SNP panels and 0.70 for the 48-SNP panels. The average probability of correct assignment was higher using simulated individuals (*f*_ORCA_) for both the 96-SNP panel (0.96) and the 48-SNP panel (0.85).

**Table 4:** P-values from post hoc Tukey's Honestly Significant Difference test for comparisons of performance of 96- and 48- SNP panel using empirical data.

	96 F_ST_	96 I_n_	96 LC	96 BELS	96 WL	96 Random	96 AVG	48 F_ST_	48 I_n_	48 LC	48 BELS	48 WL	48 Random
96 I_n_	**1.00**												
96 LC	**1.00**	**1.00**											
96 BELS	0.01	0.01	0.01										
96 WL	**1.00**	**1.00**	**1.00**	**0.05**									
96 Random	**0.93**	**0.92**	**0.93**	**0.71**	**0.99**								
96 AVG	**1.00**	**1.00**	**1.00**	0.02	**1.00**	**0.96**							
48 F_ST_	0.00	0.00	0.00	**1.00**	0.00	**0.21**	0.00						
48 I_n_	0.01	0.01	0.01	**1.00**	0.05	**0.68**	0.02	**1.00**					
48 LC	0.00	0.00	0.00	**0.99**	0.00	0.05	0.00	**1.00**	**0.99**				
48 BELS	0.00	0.00	0.00	0.00	0.00	0.00	0.00	0.00	0.00	0.00			
48 WL	0.00	0.00	0.00	0.03	0.00	0.00	0.00	**0.26**	0.04	**0.63**	0.00		
48 Random	0.00	0.00	0.00	0.00	0.00	0.00	0.00	0.00	0.00	0.00	0.00	**0.53**	
48 AVG	0.00	0.00	0.00	**0.90**	0.00	0.01	0.00	**1.00**	**0.91**	**1.00**	0.00	**0.90**	0.00

SNP panels were generated using the following measures: genetic differentiation (F_ST_), Rosenberg's informativeness (I_n_), average contribution of locus to principal components (LC), ranks from the locus selection programs BELS and WHICHLOCI (WL), average rank based on the five preceding measures, and randomly generated ranks. Non-significant p-values are indicated in bold.

**Table 5:** P-values from post hoc Tukey's Honestly Significant Difference test for comparisons of performance of 96- and 48- SNP panel using simulated data.

	96 F_ST_	96 I_n_	96 LC	96 BELS	96 WL	96 Random	96 AVG	48 F_ST_	48 I_n_	48 LC	48 BELS	48 WL	48 Random
96 I_n_	**1.00**												
96 LC	**0.77**	**1.00**											
96 BELS	0.00	0.00	0.00										
96 WL	**0.28**	**0.83**	**1.00**	0.00									
96 Random	0.00	0.00	0.00	0.00	0.00								
96 AVG	0.02	**0.24**	**0.95**	0.00	**1.00**	0.00							
48 F_ST_	0.00	0.00	0.00	0.00	0.00	0.00	0.00						
48 I_n_	0.00	0.00	0.00	0.00	0.00	0.00	0.00	0.00					
48 LC	0.00	0.00	0.00	0.00	0.00	0.00	0.00	0.00	0.00				
48 BELS	0.00	0.00	0.00	0.00	0.00	0.00	0.00	0.00	0.00	0.00			
48 WL	0.00	0.00	0.00	0.00	0.00	0.00	0.00	0.00	0.00	0.00	0.00		
48 Random	0.00	0.00	0.00	0.00	0.00	0.00	0.00	0.00	0.00	0.00	0.00	0.00	
48 AVG	0.00	0.00	0.00	0.00	0.00	0.00	0.00	0.00	0.00	0.00	0.00	0.00	0.00

SNP panels were generated using the following measures: genetic differentiation (F_ST_), Rosenberg's informativeness (I_n_), average contribution of locus to principal components (LC), ranks from the locus selection programs BELS and WHICHLOCI (WL), average rank based on the five preceding measures, and randomly generated ranks. Non-significant p-values are indicated in bold.

Most of the 96-SNP panels performed similar to and significantly better than the 48-SNP panels (P<0.001) except for the BELS and randomly generated 96-SNP panels; these performed similar to the 48-SNP F_ST_ and I_n_ panels when using the empirical data (Table 4). The 48-SNP panels tested empirically performed similarly to at least one other 48-SNP panel (Table 4) except for the BELS panel which had the lowest average probability of correct assignment (0.49). All of the 48-SNP panels performed differently (P<0.001) using the simulated data (Table 5). The F_ST_, I_n_, and LC panels had the highest average probability of correct assignment (0.87–0.88) and the randomly generated panel (0.84) and the BELS panel (0.72) had the lowest average.

**Figure 9:**
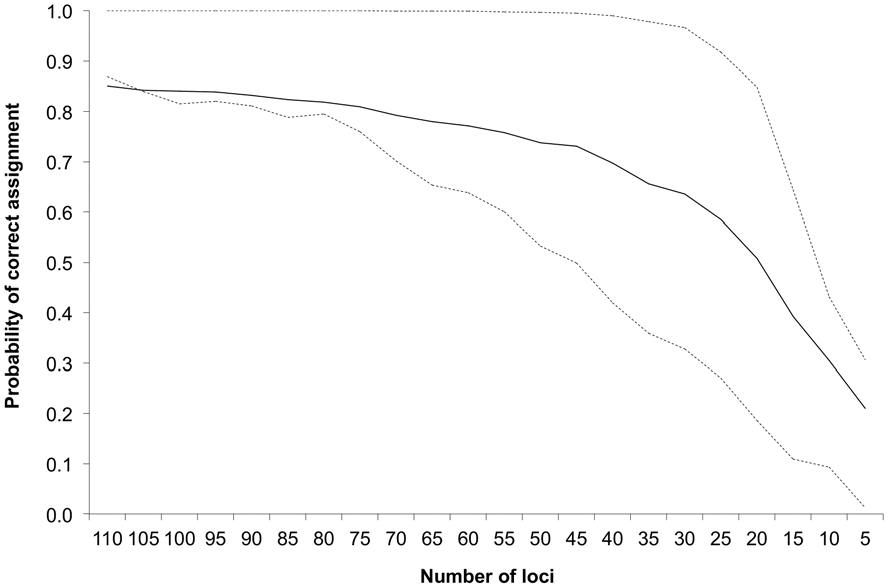
Probability of correct assignment with decreasing number of loci. Average probability of correct assignment (solid line) is flanked by 1^st^ and 3^rd^ quartiles (dashed lines). Loci were removed five at a time by lowest rank.

The average probability of correct assignment was 0.85 when all 110 polymorphic loci were used for individual assignment. The average probability of correct assignment decreased as loci were removed but remained above 0.7 until only 40 loci remained (Figure 9). The range of probabilities for correct assignment also increased as loci were removed from the data set. The 1^st^ quartile nearly flanks the average probability of correct assignment until only 75 loci remain and is higher than the mean in some cases where the median of the 25^th^ percentile data is actually higher than data mean. The probability of correct assignment at the 75^th^ quantile remained nearly as high as 1.0 for some individuals until only 30 loci remain (Figure 9). The greatest changes in assignment accuracy began when dropping from 20 loci (0.85) to 15 loci (0.64).

## Discussion

### SNP discovery

Our goal was to expand the battery of 45 commonly used SNPs into sets of 48 or 96 to better utilize the medium density arrays commonly in use for sockeye salmon. We successfully developed and validated 43 new SNP assays. Using HRMA, we were able to quickly and affordably evaluate putative SNPs. Putative SNPs were eliminated if HRMA revealed that there was no SNP present in the amplified region or if there were multiple variants (suggesting paralogous variation). Unsuccessfully amplified loci may have been adjacent to intron-exon boundaries resulting in PCR failure. This source of putative SNP drop-out is difficult to avoid when using transcriptome without a reference genome to identify intron-exon boundaries, and the lack of a reference genome may continue to present challenges for future SNP discovery. However, despite these challenges, improved NGS technologies and improved bioinformatics will continue to accelerate SNP discovery in non-model organisms [10]. One drawback of our approach was that sequence assembly using short reads and transcriptome sequences, especially without a reference genome, was difficult and computationally exhaustive. Some false positives, especially in the SOLiD-derived transcriptome, were probably dependent on the method of assembling the short reads [38]. We no longer use SOLiD sequencing for SNP discovery because these problems are exacerbated in duplicated salmonids.

One facet of SNP discovery that warrants attention is ascertainment bias which is introduced during the SNP discovery process because the variation being captured is usually only representative of a small number of individuals [8], [52], [53]. Concerns about ascertainment bias have been previously addressed (e.g. [54]), and there appears to be a growing consensus that the effects of ascertainment bias are nearly negligible when parsing out relationships between populations when more SNPs are used [55], [56]. In this study, ascertainment bias for some SNPs would have been introduced during the initial SNP detection step where sequences from only a few individuals in Bristol Bay, Alaska, were used. However, using populations across the species' range for SNP validation was meant to ensure the capture of SNPs to resolve Bristol Bay populations while also providing geographically broad resolution. Despite a limited number of ascertainment fish, there does not appear to be a strong signal for ascertainment bias in this study. Allelic richness, which can be a signal of ascertainment bias, does not vary much across the range of populations surveyed. The significant variation among regions may reflect underlying differences in genetic diversity between populations as there is no clear geographic trend in mean allelic richness.

### SNP assessment

Most populations were easily differentiated except for the Main Bay Hatchery-Coghill Lake pair in Southcentral Alaska. The exception may be attributed to the fact that fertilized eggs from Coghill Lake fish were introduced into the Main Bay Hatchery population during the last three decades (PWSAC Hatcheries, www.pwsac.com/mbh.htm). The high F_ST_ values observed between all other populations and regions in this study reflect the large geographic range surveyed in addition to the extreme philopatry of the species which results in strong genetic differences across even small geographic scales [57]. Over 40% of observed genetic variation is accounted for in the first principal component of genetic distance, which differentiates Washington, British Columbia, Southcentral Alaska, and the more western collections. The second principal component primarily differentiates among the western collections: Kamchatka, Bristol Bay and the Alaska Peninsula. This suggests that there are different suites of SNPs that are better for resolving population structure across different geographical scales (e.g., [17]). One approach to identifying an additional suite of SNPs would be to rank loci by their contribution only to a specific principal component that differentiates populations of interest.

Linkage disequilibrium was observed in some loci and in some locations, but only between loci where linkage relationships or linkage disequilibrium were noted in other studies (e.g., the MHC SNPs [24]). The treatment of linked loci is often dependent on the application and decided by the primary investigator. Often combining linked loci can provide increased resolution [58]; however some software used for genetic analyses such as population assignment cannot use this phased data which consists of multi-allelic haplotypes. In some cases, linked loci appear to provide similar information, measuring the same allele frequencies across populations. Although these loci may have similar resolving power, they may only provide redundant information (i.e. providing the power to differentiate between the same populations) in which case one locus might be dropped from the loci set without losing resolution. Developing more standardized methods for parsing the difference between the value of a locus for its resolving power and its value due to uniqueness of information will become important for creating highly optimized SNP panels.

In previous studies, using a subset of these SNPs, the MHC loci have often been identified as candidates for natural selection [23], [24], [51], [58]; however, that was not the case here. Those studies surveyed populations across a much smaller geographic range [58] and for different life history types [24] suggesting that the MHC loci might be displaying a signature of local adaptation. In this study, strong genetic differentiation across a large geographic range may dwarf a signal of selection at the MHC loci that may occur at smaller geographic scales.

Studies have shown that candidate loci can greatly improve the resolution of population structure [46], [47] and the accuracy of individual assignment (e.g., [51], [59]). This warrants the exploratory use of these methods for locus assessment. Many of the outlier loci were also some of the most informative; the added value of including these loci was demonstrated most recently by Ackerman et al. [51] where the inclusion of these non-neutral markers significantly improved individual assignment. Here we found that the removal of outlier loci did significantly decrease F_ST_ values, but the relationships between populations remained the same. In studies where there is less natural variation between populations, the value of including outlier loci in individual assignment would most likely be higher [60]. Despite concerns regarding the influence of these markers in population genetic studies, it is evident that non-neutral markers are valuable for population identification.

### SNP ranking

SNPs can be ranked in a variety of ways. Computer programs such as WHICHLOCI generate optimized SNP panels using genetic data, rigorous statistical algorithms, and general objective functions. Alternatively, ranking procedures developed for specific applications might consider everything from laboratory performance to accuracy of individual assignment. Unsurprisingly, sample size does impact ranking as we observed greater variation in locus ranks using the training set (Figure 6), which had half as many individuals as the full assessment populations. However, many of the highest ranking loci remained highest ranking loci (e.g. *One_apoe-83*). Interestingly, differences in locus ranks based on diversity indices (F_ST_, I_n_, and LC) versus the likelihood-based ranking programs BELS and WHICHLOCI were greater using the training set. Sample size may have a greater impact on ranking when using these programs. Although the ranking strategies used here are not novel, we believe that showing a comparison of ranking approaches for the same data is informative and will be of value once researchers have access to hundreds of SNPs.

### Panel testing

Increasing the panel size from 48 to 96 significantly improved individual assignment (Figure 8); when loci were dropped sequentially by rank, correct assignment remained somewhat level until about panel size 30 and then markedly dropped (Figure 9). More interestingly, it appears that as the number of available loci increases, the ranking approach will become more important as evidenced by differential performance of the 48-SNP panels using both empirical and simulated data (Tables 4, 5). One would expect that, if we were creating a 96-SNP panel from over 200 markers, we would see more substantial differences in panel performance.

Testing panel performance with both empirical and simulated data yielded slightly different results. The greater variation in probability of correct assignment observed using empirical data may be partially attributed to individual differences in DNA quality. Some of the samples may have suffered tissue and DNA degradation; missing genotypes in these increase assignment difficulty. With simulated individuals there no variation in data quality, explaining the low variance and higher average probability of correct assignment. Simulated data provide a better idea of which panel performs best based solely on SNP composition because sample quality is not a source of variation; empirical data provides a better idea of panel performance in an actual study. There would be less variation in the probability of correct assignment with larger sample sizes for SNP ranking (training set) and evaluation (holdout set).

There was a pattern in panel performance for both panel-testing approaches and for panel sizes. F_ST_, I_n_, and LC panels were often the most similar and had the highest average probability of correct assignment. The similarity between these panels is expected since these ranking methods are all highly correlated (e.g. Figure 5). These three panels were also similar to the WHICHLOCI panel and the panel based on average locus rank across all five ranking methods (Table 4, 5). The BELS panels had the lowest average probability of correct assignment which was even lower than the panel of randomly selected loci. BELS has difficulty ranking loci when assignment accuracy is set to be 100% [17], possibly accounting for the panel's poor performance. Despite the poor performance of the BELS panels, there is continuity in how BELS ranked loci; the highest ranked loci remained the highest ranked over multiple runs (Figure 7). Some of the highest ranked loci were also highly ranked for F_ST_, I_n_, and LC (e.g. *One_apoe-83*). The stability of highest locus ranks and variability of mid- and low-performing locus ranks might be an artifact of the program's intent to determine a minimum set of loci that maximizes performance. Once the best performing loci, for example the top 40, have been identified, the addition or removal of the remaining loci results in minimal changes in performance resulting in arbitrary ranks.

### Conclusions

The popularity of a given type of molecular marker has changed repeatedly over recent history. Regardless of the marker type or discovery method, there is continued interest in developing methods for ranking and evaluating markers, hence the design of locus selection programs such as BELS and WHICHLOCI. SNPs have recently become a marker of choice for several non-model species, and there is growing interest in methods to evaluate the ever-increasing number of SNPs. Here we not only describe an effective method for SNP discovery in the culturally and commercially important non-model sockeye salmon, but we also demonstrate how common locus-ranking methods perform differently when developing a SNP panel. Although our investigations explore the role of loci for use across a large geographic scale with high overall differentiation, the same approach can be applied and optimized for finer geographic resolution. The steps outlined here provide a starting place for developing a minimum panel size for maximum assignment accuracy for any specific system or question. Here we recommend panels of 48 or 96 SNPs that will expand the options for improved management and conservation of the iconic sockeye salmon.

## Supporting Information

Table S1 All 114 loci in alphabetic order with descriptive statistics (Ho, He, & F_ST_) for 12 SNP-assessment populations. Average rank based on training set individuals for five ranking approaches: genetic differentiation (F_ST_), Rosenberg's informativeness (I_n_), average contribution of locus to principal components (LC), and ranks from the locus selection programs BELS and WHICHLOCI. The numeral 1 indicates the highest rank. Locus ranks for each approach are based on a training set of the 12 SNP-assessment populations.
(DOCX)

Table S2 Forward and reverse primer and probe sequences for all newly developed SNPs featured in this paper with SNP characterization and gene annotation when possible.
(DOC)
