# Supplementary material for: Rank and Order: Evaluating the Performance of SNPs for Individual Assignment in a Non-Model Organism
Source: PLoS One. 2012 Nov 20;7(11):e49018. doi: 10.1371/journal.pone.0049018 (PMC3502385; doi:10.1371/journal.pone.0049018)
Supplement: Table S1 — All 114 loci in alphabetic order with descriptive statistics (Ho, He, & FST) for 12 SNP-assessment populations. Average rank based on training set individuals for five ranking approaches: genetic differentiation (FST), Rosenberg's informativeness (In), average contribution of locus to principal components (LC), and ranks from the locus selection programs BELS and WHICHLOCI. The numeral 1 indicates the highest rank. Locus ranks for each approach are based on a training set of the 12 SNP-assessment populations. (DOCX) [file pone.0049018.s001.docx]

Table S1. All 114 loci in alphabetic order with descriptive statistics (Ho, He, & FST) for full 12 SNP-assessment populations. Average rank based on training set individuals and five ranking approaches: genetic differentiation (F_ST_), Rosenberg's informativeness (I_n_), average contribution of locus to principal components (LC), and ranks from the locus selection programs BELS and WHICHLOCI. The numeral 1 indicates the highest rank. Locus ranks for each approach are based on a training set of the 12 SNP-assessment populations.

| Avg. Rank | Locus name | Reference | H_o_ | H_e_ | F_ST_ | F_ST_ Rank | I_n_ Rank | LC Rank | BELS Rank | WHICHLOCI Rank |
| --- | --- | --- | --- | --- | --- | --- | --- | --- | --- | --- |
| 43 | *One_ACBP-79* | Elfstrom et al. 2006 | 0.41 | 0.41 | 0.126 | 40 | 33 | 47 | 73 | 46 |
| 18 | *One_agt-132* | this study | 0.40 | 0.39 | 0.231 | 10 | 9 | 9 | 103 | 38 |
| 106 | *One_aldB-152* | Campbell & Narum 2011 | 0.31 | 0.32 | 0.021 | 102 | 101 | 93 | 29 | 107 |
| 99 | *One_ALDOB-135* | Elfstrom et al. 2006 | 0.25 | 0.25 | 0.032 | 87 | 80 | 88 | 54 | 91 |
| 2 | *One_apoe-83* | this study | 0.34 | 0.34 | 0.316 | 4 | 1 | 2 | 1 | 35 |
|  | *One_bckB-137* | Campbell & Narum 2011 | monomorphic | | | | | | | |
| 90 | *One_c3-98* | this study | 0.10 | 0.09 | 0.015 | 104 | 103 | 98 | 46 | 34 |
|  | *One_ccd16-131* | this study | monomorphic | | | | | | | |
| 47 | *One_CD9-269* | this study | 0.32 | 0.34 | 0.086 | 54 | 63 | 41 | 32 | 70 |
| 74 | *One_cetn1-167* | this study | 0.43 | 0.43 | 0.100 | 55 | 55 | 61 | 84 | 84 |
| 12 | *One_CFP1* | DFO | 0.28 | 0.28 | 0.204 | 20 | 18 | 18 | 47 | 45 |
| 104 | *One_cin-177* | Campbell & Narum 2011 | 0.45 | 0.47 | 0.051 | 75 | 74 | 103 | 87 | 85 |
| 55 | *One_ctgf-301* | Elfstrom et al. 2006 | 0.06 | 0.06 | 0.057 | 88 | 95 | 82 | 13 | 12 |
| 63 | *One_dds-529* | Campbell & Narum 2011 | 0.45 | 0.43 | 0.084 | 74 | 76 | 49 | 30 | 81 |
| 107 | *One_DDX5-86* | this study | 0.43 | 0.45 | 0.019 | 103 | 104 | 108 | 28 | 108 |
| 95 | *One_E2-65* | Smith et al. 2005 | 0.25 | 0.24 | 0.053 | 91 | 86 | 76 | 44 | 95 |
| 70 | *One_gadd45-269* | Campbell & Narum 2011 | 0.00 | 0.00 | 0.000 | 109 | 109 | 106 | 2 | 1 |
| 86 | *One_gdh-212* | Campbell & Narum 2011 | 0.48 | 0.46 | 0.060 | 71 | 69 | 85 | 66 | 75 |
| 24 | *One_GHII-2165* | Elfstrom et al. 2006 | 0.26 | 0.26 | 0.226 | 12 | 14 | 10 | 100 | 44 |
| 82 | *One_ghsR-66* | Campbell & Narum 2011 | 0.37 | 0.38 | 0.082 | 66 | 54 | 77 | 80 | 76 |
| 101 | *One_GPDH-201* | Smith et al. 2005 | 0.46 | 0.47 | 0.043 | 85 | 87 | 97 | 52 | 98 |
| 66 | *One_GPDH2-187* | Smith et al. 2005 | 0.23 | 0.22 | 0.084 | 53 | 48 | 58 | 86 | 71 |
| 10 | *One_GPH-414* | Elfstrom et al. 2006 | 0.36 | 0.36 | 0.218 | 9 | 7 | 8 | 85 | 36 |
| 57 | *One_HGFA-49* | Smith et al. 2005 | 0.32 | 0.34 | 0.065 | 65 | 73 | 43 | 37 | 74 |
| 73 | *One_HpaI-71* | Elfstrom et al. 2006 | 0.43 | 0.44 | 0.103 | 58 | 58 | 74 | 74 | 72 |
| 8 | *One_HpaI-99* | Elfstrom et al. 2006 | 0.24 | 0.23 | 0.366 | 2 | 2 | 3 | 89 | 37 |
| 108 | *One_hsc71-220* | Elfstrom et al. 2006 | 0.35 | 0.34 | 0.037 | 92 | 88 | 94 | 83 | 101 |
| 92 | *One_Hsp47* | DFO | 0.28 | 0.29 | 0.062 | 94 | 94 | 72 | 39 | 89 |
| 48 | *One_Ig-90* | this study | 0.10 | 0.09 | 0.062 | 67 | 70 | 62 | 45 | 17 |
| 93 | *One_IL8r-362* | Habicht et al. 2010 | 0.15 | 0.14 | 0.049 | 83 | 79 | 87 | 36 | 103 |
| 71 | *One_ins-107* | Smith et al. 2005 | 0.46 | 0.45 | 0.073 | 81 | 75 | 59 | 53 | 59 |
| 37 | *One_KCT1-453* | this study | 0.30 | 0.29 | 0.120 | 37 | 53 | 29 | 35 | 61 |
| 105 | *One_KPNA-422* | Elfstrom et al. 2006 | 0.33 | 0.33 | 0.018 | 106 | 106 | 104 | 10 | 104 |
|  | *One_LEI-87* | Elfstrom et al. 2006 | monomorphic | | | | | | | |
| 44 | *One_leptin-92* | Campbell & Narum 2011 | 0.45 | 0.45 | 0.088 | 50 | 45 | 46 | 72 | 31 |
| 89 | *One_lpp1-44* | this study | 0.32 | 0.31 | 0.059 | 79 | 77 | 70 | 61 | 90 |
| 64 | *One_MARCKS-241* | Habicht et al. 2010 | 0.04 | 0.04 | 0.029 | 100 | 102 | 95 | 5 | 10 |
| 6 | *One_metA-253* | Campbell & Narum 2011 | 0.13 | 0.14 | 0.204 | 16 | 16 | 19 | 69 | 8 |
| 60 | *One_MHC2_190* | Elfstrom et al. 2006 | 0.40 | 0.41 | 0.131 | 45 | 42 | 79 | 90 | 51 |
| 28 | *One_MHC2_251* | Elfstrom et al. 2006 | 0.35 | 0.35 | 0.204 | 13 | 11 | 22 | 104 | 40 |
| 59 | *One_Mkpro-129* | Campbell & Narum 2011 | 0.47 | 0.46 | 0.059 | 70 | 62 | 56 | 38 | 79 |
| 72 | *One_ODC1-196* | this study | 0.44 | 0.45 | 0.058 | 73 | 72 | 55 | 57 | 77 |
| 20 | *One_Ots213-181* | Elfstrom et al. 2006 | 0.31 | 0.31 | 0.158 | 24 | 25 | 28 | 42 | 55 |
| 26 | *One_p53-534* | Elfstrom et al. 2006 | 0.09 | 0.08 | 0.111 | 35 | 47 | 38 | 56 | 9 |
| 78 | *One_parp3-170* | Campbell & Narum 2011 | 0.01 | 0.01 | 0.026 | 108 | 108 | 107 | 20 | 2 |
| 45 | *One_PIP* | DFO | 0.45 | 0.45 | 0.092 | 43 | 41 | 40 | 76 | 48 |
| 5 | *One_ppie-74* | Campbell & Narum 2011 | 0.10 | 0.11 | 0.364 | 3 | 5 | 4 | 101 | 7 |
| 38 | *One_Prl2* | Elfstrom et al. 2006 | 0.41 | 0.43 | 0.158 | 27 | 19 | 34 | 93 | 43 |
| 46 | *One_psme2-354* | this study | 0.30 | 0.31 | 0.102 | 48 | 35 | 52 | 59 | 63 |
| 102 | *One_rab1a-76* | this study | 0.20 | 0.20 | 0.044 | 99 | 99 | 100 | 15 | 106 |
| 3 | *One_RAG1-103* | Elfstrom et al. 2006 | 0.08 | 0.07 | 0.166 | 21 | 44 | 12 | 3 | 14 |
| 67 | *One_RAG3-93* | Elfstrom et al. 2006 | 0.17 | 0.17 | 0.064 | 52 | 50 | 48 | 81 | 87 |
| 49 | *One_redd1-414* | Campbell & Narum 2011 | 0.44 | 0.45 | 0.089 | 69 | 68 | 51 | 7 | 68 |
| 52 | *One_RFC2-102* | Smith et al. 2005 | 0.30 | 0.30 | 0.113 | 39 | 30 | 37 | 98 | 69 |
| 79 | *One_RFC2-285* | Smith et al. 2005 | 0.07 | 0.06 | 0.043 | 95 | 91 | 91 | 50 | 20 |
| 84 | *One_RH2op-395* | Elfstrom et al. 2006 | 0.02 | 0.02 | 0.006 | 107 | 107 | 109 | 25 | 11 |
| 103 | *One_rpo2j-261* | Campbell & Narum 2011 | 0.28 | 0.29 | 0.030 | 98 | 96 | 102 | 22 | 105 |
| 76 | *One_sast-211* | Campbell & Narum 2011 | 0.08 | 0.09 | 0.058 | 76 | 83 | 101 | 51 | 30 |
| 75 | *One_serpin-75* | Smith et al. 2005 | 0.03 | 0.03 | 0.013 | 105 | 105 | 96 | 12 | 21 |
| 80 | *One_spf30-207* | Campbell & Narum 2011 | 0.30 | 0.30 | 0.056 | 82 | 85 | 90 | 4 | 86 |
| 25 | *One_srp09-127* | Campbell & Narum 2011 | 0.13 | 0.14 | 0.100 | 47 | 49 | 44 | 14 | 29 |
| 88 | *One_ssrd-135* | Campbell & Narum 2011 | 0.48 | 0.48 | 0.038 | 90 | 93 | 81 | 9 | 96 |
| 15 | *One_STC-410* | Elfstrom et al. 2006 | 0.29 | 0.31 | 0.268 | 6 | 3 | 6 | 110 | 28 |
| 21 | *One_STR07* | Elfstrom et al. 2006 | 0.37 | 0.37 | 0.220 | 7 | 6 | 15 | 107 | 39 |
| 30 | *One_SUMO1-6* | Campbell & Narum 2011 | 0.28 | 0.30 | 0.144 | 28 | 21 | 27 | 62 | 54 |
| 17 | *One_sys1-230* | Campbell & Narum 2011 | 0.40 | 0.38 | 0.258 | 11 | 8 | 7 | 106 | 33 |
| 36 | *One_taf12-248* | Campbell & Narum 2011 | 0.07 | 0.07 | 0.107 | 49 | 51 | 39 | 68 | 6 |
| 19 | *One_Tf_ex11-750* | Elfstrom et al. 2006 | 0.36 | 0.39 | 0.148 | 29 | 24 | 32 | 41 | 47 |
| 42 | *One_Tf_in3-182* | Elfstrom et al. 2006 | 0.14 | 0.14 | 0.132 | 30 | 26 | 63 | 105 | 13 |
| 109 | *One_tshB-92* | Campbell & Narum 2011 | 0.09 | 0.09 | 0.022 | 101 | 100 | 105 | 43 | 109 |
| 13 | *One_txnip-401* | Campbell & Narum 2011 | 0.07 | 0.07 | 0.148 | 32 | 37 | 42 | 34 | 5 |
| 110 | *One_U1002-101* | this study | 0.00 | 0.00 | 0.006 | 110 | 110 | 110 | 26 | 110 |
| 22 | *One_U1003-75* | this study | 0.28 | 0.29 | 0.275 | 8 | 4 | 5 | 109 | 50 |
| 65 | *One_U1004-183* | this study | 0.38 | 0.39 | 0.104 | 51 | 40 | 69 | 94 | 58 |
| 77 | *One_U1009-91* | this study | 0.31 | 0.32 | 0.068 | 64 | 65 | 65 | 65 | 82 |
| 1 | *One_U1010-81* | this study | 0.11 | 0.10 | 0.319 | 1 | 10 | 1 | 8 | 22 |
| 85 | *One_U1012-68* | this study | 0.29 | 0.29 | 0.099 | 63 | 56 | 73 | 88 | 80 |
| 83 | *One_U1013-108* | this study | 0.23 | 0.23 | 0.053 | 72 | 71 | 67 | 64 | 83 |
| 100 | *One_U1014-74* | this study | 0.20 | 0.19 | 0.035 | 93 | 89 | 99 | 23 | 102 |
| 39 | *One_U1016-115* | this study | 0.40 | 0.41 | 0.134 | 38 | 34 | 31 | 63 | 53 |
| 4 | *One_U1017-62* | this study | 0.07 | 0.07 | 0.219 | 18 | 31 | 16 | 49 | 4 |
| 27 | *One_U1024-197* | this study | 0.24 | 0.24 | 0.126 | 36 | 39 | 36 | 17 | 60 |
| 54 | *One_U1101* | this study | 0.35 | 0.35 | 0.079 | 68 | 66 | 50 | 48 | 57 |
| 16 | *One_U1102-220* | this study | 0.20 | 0.15 | 0.178 | 17 | 22 | 20 | 79 | 23 |
| 34 | *One_U1103* | this study | 0.05 | 0.05 | 0.083 | 44 | 64 | 54 | 24 | 18 |
| 56 | *One_U1104-138* | this study | 0.02 | 0.02 | 0.049 | 84 | 98 | 86 | 19 | 3 |
| 35 | *One_U1105* | this study | 0.27 | 0.29 | 0.171 | 15 | 15 | 26 | 97 | 56 |
| 97 | *One_U1201-492* | this study | 0.49 | 0.48 | 0.044 | 97 | 97 | 78 | 21 | 100 |
| 58 | *One_U1202-1052* | this study | 0.35 | 0.34 | 0.086 | 60 | 52 | 45 | 70 | 73 |
| 68 | *One_U1203-175* | this study | 0.39 | 0.39 | 0.094 | 56 | 57 | 53 | 92 | 64 |
| 41 | *One_U1204-53* | this study | 0.29 | 0.30 | 0.125 | 25 | 20 | 24 | 102 | 65 |
| 61 | *One_U1205-57* | this study | 0.08 | 0.08 | 0.083 | 61 | 78 | 89 | 60 | 19 |
| 91 | *One_U1206-108* | this study | 0.31 | 0.32 | 0.034 | 96 | 92 | 92 | 6 | 99 |
| 31 | *One_U1208-67* | this study | 0.37 | 0.40 | 0.129 | 42 | 38 | 25 | 27 | 62 |
| 23 | *One_U1209-111* | this study | 0.19 | 0.17 | 0.250 | 5 | 12 | 11 | 108 | 42 |
| 53 | *One_U1210-173* | this study | 0.14 | 0.14 | 0.052 | 86 | 82 | 71 | 11 | 25 |
| 50 | *One_U1211-97* | this study | 0.14 | 0.14 | 0.068 | 80 | 67 | 66 | 31 | 24 |
| 51 | *One_U1212-106* | this study | 0.42 | 0.44 | 0.103 | 41 | 32 | 68 | 95 | 32 |
| 87 | *One_U1214-107* | this study | 0.17 | 0.18 | 0.074 | 62 | 60 | 83 | 71 | 92 |
| 32 | *One_U1215-82* | this study | 0.42 | 0.43 | 0.154 | 26 | 23 | 17 | 96 | 41 |
| 98 | *One_U1216-230* | this study | 0.45 | 0.47 | 0.058 | 78 | 81 | 75 | 67 | 97 |
| 96 | *One_U301-92* | Elfstrom et al. 2006 | 0.24 | 0.24 | 0.066 | 77 | 84 | 80 | 58 | 93 |
| 11 | *One_U401-224* | Habicht et al. 2010 | 0.45 | 0.44 | 0.117 | 31 | 27 | 23 | 16 | 49 |
| 7 | *One_U404-229* | Habicht et al. 2010 | 0.14 | 0.13 | 0.136 | 33 | 28 | 35 | 18 | 16 |
| 33 | *One_U502-167* | Habicht et al. 2010 | 0.09 | 0.10 | 0.129 | 34 | 43 | 33 | 78 | 15 |
| 62 | *One_U503-170* | Habicht et al. 2010 | 0.28 | 0.29 | 0.089 | 46 | 46 | 57 | 91 | 67 |
| 94 | *One_U504-141* | Habicht et al. 2010 | 0.35 | 0.37 | 0.041 | 89 | 90 | 84 | 33 | 94 |
| 29 | *One_U508-533* | Habicht et al. 2010 | 0.10 | 0.12 | 0.183 | 22 | 29 | 21 | 40 | 78 |
| 81 | *One_vamp5-255* | Campbell & Narum 2011 | 0.28 | 0.29 | 0.108 | 59 | 61 | 64 | 75 | 88 |
| 14 | *One_vatf-214* | Campbell & Narum 2011 | 0.11 | 0.11 | 0.178 | 19 | 36 | 13 | 55 | 27 |
| 69 | *One_VIM-569* | Elfstrom et al. 2006 | 0.25 | 0.24 | 0.105 | 57 | 59 | 60 | 82 | 66 |
|  | *One_zn706-68* | Campbell & Narum 2011 | monomorphic | | | | | | | |
| 40 | *One_ZNF-61* | Habicht et al. 2010 | 0.35 | 0.36 | 0.160 | 23 | 17 | 30 | 99 | 52 |
| 9 | *One_zP3b-49* | Smith et al. 2005 | 0.27 | 0.27 | 0.183 | 14 | 13 | 14 | 77 | 26 |
