# Supplementary material for: Rank and Order: Evaluating the Performance of SNPs for Individual Assignment in a Non-Model Organism
Source: PLoS One. 2012 Nov 20;7(11):e49018. doi: 10.1371/journal.pone.0049018 (PMC3502385; doi:10.1371/journal.pone.0049018)
Supplement: Table S2 — Forward and reverse primer and probe sequences for all newly developed SNPs featured in this paper with SNP characterization and gene annotation when possible. (DOC) [file pone.0049018.s002.doc]

Table S2. Forward and reverse primer and probe sequences for all newly developed SNP markers featured in this paper with SNP characterization and gene annotation when possible.

| Locus name | NCBI accession | Gene | E-value | VIC/  FAM | Primer sequences | Probe sequences |
| --- | --- | --- | --- | --- | --- | --- |
| *One_agt-132* | 445261120 | Angiotensinogen (angt) | 5.0E-70 | A/C | F:GACCCAGATCAACAACTTCATCCA R:TGGTTGAGCTAAGGTCCTTGAAC | VIC: ACAGGAAAATCACGAGCCT FAM:CAGGAAAATCCCGAGCCT |
| *One_apoe-83* | 445261132 | apolipoprotein E (apoe) | 1.0E-46 | C/T | F:CGCCATGGACAAGGTCAAG R:GGCACAGTGCTTCCAAACC | VIC:TTTAGACGGCGGTCTC FAM:ATTTAGACAGCGGTCTC |
| *One_c3-98* | 445261138 | complement component C3 | 1.0E-75 | C/T | F:GAGTGTGGAACTGGTTCTTGTTG R:GCCGGCAGGGCATCA | VIC:GTTGATGGACCACCTGGT FAM:TTGATGGACCACTTGGT |
| *One_ccd16-131* | 445261145 | Coiled-coil domain-containing protein | 7.0E-23 | C/T | F:CCGTGACCTGTTGAACTTTGTTTAG R:TCACGTTCTTGGAAAACAGC | VIC:AAGGAGAAAGTTGCCGAGCT FAM:ATAAGGAGAAAGTTACCGAGCT |
| *One_CD9-269* | 445261152 | similar to CD9 antigen | 1.0E-41 | C/T | F:ACGCTCTGAGGTGATATGAAACAC R:CATCCGACGTCAACATCCAAAC | VIC:TGGAATGGAGAAATC FAM:ATGGAATGAAGAAATC |
| *One_cetn1-167* | 445261159 | Centrin-1 (cetn1) | 5.0E-56 | A/C | F:CAGAAATCCTGACTGTTAAAACAATGCA R:CTGCTCGTTGATCTCTCCATCTC | VIC:TTGACGAAGCAGACCGA FAM:TTGACGAAGCCGACCGA |
| *One_DDX5-86* | 445261166 | Probable ATP-dependent RNA helicase | 2.0E-72 | C/T | F:CTCCCACATTGATCTGGACGTA R:TGCCACTTGGCCCAAAGAG | VIC:AGGACTTCCTGAAGGAC FAM:AGGACTTCCTAAAGGAC |
| *One_Ig-90* | 445261172 | Ig kappa chain V region K29-213 | 3.0E-11 | C/G | F:GGATTGTGGTAACTCTGACAGTAGT R:CATCTAAATTCAGTGGCAGTGGGTTA | VIC:CTCCTGCATCTTCAGCC FAM:CCTGCATGTTCAGCC |
| *One_KCT1-453* | 445261179 | 3-ketoacyl-CoA thiolase, mitochondrial | 2.0E-58 | G/T | F:GGGAAAGTATGCTGTGGGATCAG R:GGTTCCTCAGTGAGTGTTCTCTATG | VIC:TGGTCAGGGTATCGCCATA FAM:TGGTCAGGGTATCTCCATA |
| *One_lpp1-44* | 445261186 | Lipid phosphate phosphohydrolase 1 | 2.0E-38 | C/T | F:GGTCCAATAGGGAGCTCAGACA R:GGGAATGAACCAGACATGTGAATG | VIC:TTGTGCTTTCCTGACCTAT FAM:TTGTGCTTTCCTAACCTAT |
| *One_ODC1-196* | 445261193 | ODC1 ornithine decarboxylase gene | 1.0E-121 | C/T | F:AACTCTGCGTCTGTCTGCTT R:TCAGATGGTTCATTATGACAGCAACA | VIC:CGAACAGGGCTGGATG FAM:CGAACAGGACTGGATG |
| *One_psme2-354* | 445261200 | Proteasome activator complex subunit 2 | 5.0E-14 | A/G | F:TGGTCCTTCAGGTACTTTTCAGAGA R:CAAATGCCAATTCTCACCACATGA | VIC:TGATGCAGTAGCTAAAG FAM:ATGCAGTGGCTAAAG |
| *One_rab1a-76* | 445261207 | no hits |  | G/T | F:TCGCCATATTCTCTCTCCCTATCC R:ATCCACTCAGACCCATATCTACCAA | VIC:TGTGGAGCAAGGTAACT FAM:TGTGGAGCAATGTAACT |
| *One_U1002-101* | 445261214 | no hits |  | G/T | F:GCCAACCCTATACTGTACGGATTTTT R:TCCGTTGCATTGTCCATCCA | VIC:TCGTTCCAAAGAATGTTGTG FAM:CGTTCCAAAGAATTTTGTG |
| *One_U1003-75* | 445261221 | no hits |  | C/T | F:TCACGAGCCCCAGTCAGA R:CGGGTTTCGGTGGTTTAGTATTCTA | VIC:AGAGACTACTTCCTTTTTG FAM:AGAGACTACTTCTTTTTTG |
| *One_U1004-183* | 445261228 | no hits |  | A/G | F:GGTGTGACTGCTGTGTTTAATTGC R:ACCATCATTACACAGCAATTCTGAGT | VIC:AAGTTCCCTGTATTTCTT FAM:TCCCTGCATTTCTT |
| *One_U1009-91* | 445261235 | no hits |  | A/G | F:CTCTGTCCTTGAACTGTTGTCTGTT R:GCCGCTGCTACTCTTCCT | VIC:CATGTTCTGTATGGACCC FAM:TGTTCTGTGTGGACCC |
| *One_U1010-81* | 445261242 | no hits |  | A/G | F:CAGCCCCTCGAGGTAACTG R:GTTGAGACAACAAAACGTCTACTGT | VIC:CACACCAACGTTATGTAGAG FAM:CACCAACGTTGTGTAGAG |
| *One_U1012-68* | 445261250 | no hits |  | C/T | F:TCTATTACCATACAGGCCCAGTACA R:CCTTTTGTGTCTTCCAGTCATGTGA | VIC:TGACGGGTGTTCCTGATAA FAM:TGACGGGTGTTCTTGATAA |
| *One_U1013-108* | 445261258 | no hits |  | G/T | F:TCTGTGCTCTCCTCCAGGAT R:CGAAACTGAGGAGTGCTCTGA | VIC:ACGGAATTCCTGTTGCCCT FAM:ACGGAATTCCTTTTGCCCT |
| *One_U1014-74* | 445261265 | no hits |  | C/T | F:TCCCCTGCAGCAACTGTTTT R:GGCAGAGACGGCATCCT | VIC:TTGACCTGCGCCAGTAT FAM:TTTTGACCTGCACCAGTAT |
| *One_U1016-115* | 445261272 | no hits |  | -/T | F:GGATTTTTGACTTGACCGTTTTGTGT R:ATTAACATGTGCAAAGGGAGAATGC | VIC:AATGGCAGTTTTTTATTTGA FAM:ATGGCAGTTTTTTTATTTGA |
| *One_U1017-62* | 445261279 | no hits |  | A/T | F:CAGAGAAGGACGTACCATTGATACAT R:CCGGTAGATTGGCGTTGCT | VIC:CAGAAAAACTGGTACTTGTT FAM:CAGAAAAACTGGTTCTTGTT |
| *One_U1024-197* | 445261286 | no hits |  | G/T | F:CTGAACTGATCTACCGCTCTGT R:GGAACAGATACTCCAGGAGAGATGA | VIC:ACCTGACCCAACAAA FAM:ACCTGACACAACAAA |
| *One_U1101* | 445261293 | no hits |  | C/A | F:CTATGACATGTTTATTTTAATTAGCCACCAACT R:AGTATAGCTAGGGAACCTTTCGATCTT | VIC:TGGACGTATGTCATATTT FAM:TGGACGTATGTAATATTT |
| *One_U1102-220* | 445261300 | no hits |  | C/T | F:TCCCTCTGCTGGAGAACTACAG R:GGAACAGCAGTCCTGAGTACAG | VIC:CCAGTAGTGTTTTCTG FAM:CAGTAGTGCTTTCTG |
| *One_U1103* | 445261307 | no hits |  | G/A | F:CCCAGCCGCCATGTGTA R:TGTAGTTCAGCCACCATCTTTGG | VIC:TCGGCGAAAACT FAM:TCGGCAAAAACT |
| *One_U1104-138* | 445261314 | no hits |  | G/T | F:GGAACAGAACACTGAGAATGAATGC R:GGGAATATGTCGACTGCTCACT | VIC:CCTTCTCAGAGGGTAGAGA FAM:CCTTCTCAGAGGTTAGAGA |
| *One_U1105* | 445261321 | no hits |  | T/A | F:GCCTTAATAGTGTCTTCTGATCCCTTT R:CCCTCTGTTGTCCAGACTCTTAG | VIC:CCTGTTTTTTTTAAAAGAC FAM:TCCTGTTTTTTTTTAAAGAC |
| *One_U1201-492* | 445261328 | no hits |  | A/G | F:GCTTATGACGGAGAAGAGATGCA R:AGGATACTGAAGCCCAGAGACA | VIC:AAGACTTCCTCCAGGCTC FAM:ACTTCCCCCAGGCTC |
| *One_U1202-1052* | 445261341 | no hits |  | T/C | F:CGATTTGAGTCTCCAATGGTCTCT R:ATTCCTATGGTTAACATCAATTCTATAAAGTCAT | VIC:CAAACTTTTTCATCTACATTTA FAM:ACTTTTTCATCCACATTTA |
| *One_U1203-175* | 445261348 | no hits |  | G/A | F:CCCGGAGACATACTTGATGCA R:GGAGGACCTGCAGGATCAC | VIC:CCATAGTTGCTGGGCTT FAM:CTCCATAGTTACTGGGCTT |
| *One_U1204-53* | 445261354 | no hits |  | C/T | F:GTAAAACCCTTCATGTTGGCCATT R:CTCCATGTCTGAATGTCCCATCA | VIC:ATGCATACACGCTGATGC FAM:ATGCATACACACTGATGC |
| *One_U1205-57* | 445261361 | no hits |  | A/G | F:AGTAAATGGTTATTCACGTAACGGATAAG R:CAGGACAGTTCCACATTCTAACAGA | VIC:AGTTATCATGGTCATCTCT FAM:AGTTATCATGGTCGTCTCT |
| *One_U1206-108* | 445261368 | no hits |  | G/T | F:CTGAGATGGTGCTTTCTGAGGATA R:TGGATGAAAGGGAAATTCTGTCAACA | VIC:AACATTGAGCTTCCC FAM:ATAACATTGATCTTCCC |
| *One_U1208-67* | 445261376 | no hits |  | A/C | F:ACTTGAATGTCTGTTTCGTAGGTGAT R:ACACAGTTGACAGTGGAGCAA | VIC:CCCAATGTGATTGTCAC FAM:CCAATGTGCTTGTCAC |
| *One_U1209-111* | 445261383 | no hits |  | C/T | F:GTCACGTAATCACGAGAAAGATACTAAATGT R:TCTGCGTCTCCAGAGAGGTT | VIC:CTCACATCGAGATGATC FAM:TCACATCGAAATGATC |
| *One_U1210-173* | 445261388 | no hits |  | A/G | F:ACAAAGTCTCTCTCTGAGTAGGAGTAC R:CAAAGTATCTCAGAGTGCTGATCTAGGA | VIC:CCCTCCTATTCATTATGATTGT FAM:CCTCCTATTCATTACGATTGT |
| *One_U1211-97* | 445261395 | no hits |  | C/T | F:GCGTGTCCTCCCATTAGAAGA R:CTGCAGAAGTACAGCATCTATCTGA | VIC:CTGTTTCAGTGTGCTTG FAM:CTGTTTCAGTATGCTTG |
| *One_U1212-106* | 445261402 | no hits |  | A/G | F:CGTAATGACCTACCACCATATCAGT R:TGGCATGACTTTAACAATTCCCAAAAAA | VIC:TTTTGACATACAAAAAATA FAM:TTTGACATACAGAAAATA |
| *One_U1214-107* | 445261409 | no hits |  | A/C | F:CCAAATGTACTCCATGTTGGTTAGC R:TGCCTGAGTATTAAGCTATATCATTGAAGTTTT | VIC:TAGTGACCTATTAAATTGC FAM:TGACCTATTCAATTGC |
| *One_U1215-82* | 445261416 | no hits |  | A/C | F:GTTGCTTGGTTTCGTTTGGAGTAG R:CTCCAGAAGAGGAATACCACAGTTC | VIC:AATGAGACAAAGTATTTGGT FAM:AATGAGACAAAGTCTTTGGT |
| *One_U1216-230* | 445261423 | no hits |  | A/T | F:TGGGATCGGACGTCAATAGATTTC R:GTAATACAGAGTGAGCGTGATACATTGT | VIC:CCTGGCTACTAAGTAAC FAM:CTGGCTACAAAGTAAC |
